# Screening and Stability Analysis of Reference Genes in *Pastor roseus*

**DOI:** 10.3390/genes16091056

**Published:** 2025-09-09

**Authors:** Xixiu Sun, Ran Li, Xiaojie Wang, Hongxia Hu, Kun Yang, Jianguo Wu, Jun Lin, Rong Ji, Xiaofang Ye

**Affiliations:** 1 International Research Center of Cross-Border Pest Management in Central Asia, Xinjiang Key Laboratory of Special Species Conservation and Regulatory Biology, College of Life Sciences, Xinjiang Normal University, Urumqi 830017, China; s3311827185@outlook.com (X.S.); liran00818@163.com (R.L.); wxj_971208@163.com (X.W.); huhongxia111@126.com (H.H.); 2 Research Field (Migratory Biology), Observation and Research Station of Xinjiang, Tacheng 834700, China; 16699267082@163.com; 3 Center for Grassland Biological Disaster Prevention and Control of Xinjiang Uygur Autonomous Region, Urumqi 830000, China; y1559177021@163.com (K.Y.); zhbshuju@163.com (J.W.); xjcy2009@163.com (J.L.); 4 College of Chemistry and Chemical Engineering, Changji University, Changji 831100, China

**Keywords:** *Pastor roseus*, quantitative real-time PCR, reference genes, expression stability

## Abstract

**Background/Objectives:** Optimal reference genes for normalizing RT-qPCR data depend on the species, treatments, developmental stages, and other conditions. *Pastor roseus* is a long-distance migratory bird with potential applications in locust biological control. This study applied reverse transcription quantitative PCR (RT-qPCR) to evaluate the expression stability of six genes (*RPS2*, *ACTB*, *B2M*, *SDHA*, *UBE2G2*, and *RPL4*) in blood samples from female, male, and nestling *P. roseus*. **Methods:** An integrated analysis of the expression stability of six reference genes was performed using three statistical algorithms: GeNorm, BestKeeper, and NormFinder. **Results:** The results showed that *SDHA*, *ACTB*, and *B2M* exhibited the highest expression stability among the candidate reference genes. The optimal number of reference genes was two, as determined by a pairwise variation analysis using GeNorm. Subsequent comprehensive validation using RefFinder identified *SDHA*/*ACTB* as the optimal reference gene pair for normalizing gene expression data for *P. roseus*. **Conclusions:** These findings establish a robust foundation for ensuring data accuracy in functional genomic studies of *P. roseus*.

## 1. Introduction

*P. roseus* belongs to the order Passeriformes and the family Sturnidae. It is a long-distance migratory bird that travels between Central and Western Asia, Eastern Europe, India, and Sri Lanka [1,2]. Each year, approximately 2–4 million *P. roseus* migrate to China’s Xinjiang region to breed and raise their young. They are widely distributed across locust-infested grasslands at elevations ranging from 300 to 2500 m. Within a 4–5 km radius around nesting sites, they actively prey on locusts [3]. The breeding period of *P. roseus* aligns with the peak occurrence of locust outbreaks in Xinjiang’s grasslands, thereby enabling it to function effectively as a predatory natural enemy [4]. *P. roseus* exhibits a remarkable predation capacity on locusts and demonstrates a distinct dietary specialization toward insects during its breeding season [5,6]. Adults consume 120–180 locusts daily [7], thereby safeguarding millions of hectares of grasslands in Xinjiang annually from locust infestation. Consequently, utilizing *P. roseus* for locust biological control demonstrates superior efficacy to that of alternative management measures across ecological, economic, and sustainability dimensions. Currently, research on the *P. roseus* abroad primarily focuses on physiological changes and new distribution records [8,9], such as the relationship between physiological indicators (e.g., hematocrit, plasma creatine kinase, and hemoglobin) and reproductive performance [10]. Domestic research mainly involves studies on attracting the bird and its efficacy in controlling locusts [11]. As a key species in grassland ecosystems, the ecological role of the *P. roseus* (e.g., predation on locusts) has been extensively studied; however, its physiological adaptation mechanisms, such as long-distance migration, high food consumption, and reproductive strategies, may involve complex genetic regulatory networks. Therefore, genomic research is becoming a key tool for revealing adaptive evolution and functional mechanisms. Despite its significant ecological importance, genomic studies of *P. roseus* are limited. To elucidate gene expression dynamics and the molecular basis for diverse phenotypic traits in the species, the primary prerequisite is selecting appropriate reference genes to ensure experimental rigor.

Reference genes are constitutively expressed housekeeping genes that sustain fundamental cellular activities. They exhibit stable, unvarying expression across nearly all tissue types and developmental stages, with minimal fluctuation in response to environmental variables [12]. Reference genes play a fundamental role in normalizing target gene expression levels by mitigating potential experimental errors [13]. Therefore, rigorous reference gene selection directly determines the accuracy and reproducibility of experimental results in gene expression studies. However, studies on reference genes of avians remain limited. For example, the optimal reference gene combination for *Columba livia* is *RPS2* + *18S rRNA* [12]. In *Anser anser domestica*, reference genes vary significantly among tissues (e.g., *28S rRNA* for heart, *GAPDH* for liver and ovary, *ACTB* for kidney, and *HPRT1* for muscle tissue) [14]. Wang [15] found that reference genes varied across breeds of domestic pigeon (*C. livia*), with *GluR2*, *18S rRNA*, and *RPS2* showing greater stability than those of other reference genes in meat pigeons, fancy pigeons, and racing pigeons. Screening of reference genes across different tissues in the Magang goose revealed that *ACTB* is the most suitable reference gene for the three reproductive axis tissues [16]. No single gene can guarantee a consistently stable expression under complex experimental conditions [17]. Reference genes that exhibit dynamic expression patterns are unsuitable for normalizing gene expression across different tissues, physiological states, and developmental stages [18]. Therefore, screening for appropriate reference genes across diverse sample types and experimental conditions is critical. It could ensure the stability of gene expression quantification and provide a solid foundation for subsequent functional studies, avoiding bias in target gene quantification caused by fluctuations in reference gene expression.

*P. roseus* migrates long distances to Xinjiang to feed on grassland locusts. It is able to fly thousands of kilometers in a relatively short period of time, with strong flight and metabolic abilities [19]. Despite extensive research on the breeding ecology and artificial attraction of *P. roseus*, molecular biological studies remain scarce. To elucidate the reliability of interpopulation gene expression differences, the genetic basis of migratory behavior, and key genes and molecular pathways underlying energy metabolism it is necessary to identify optimal reference genes across sexes and developmental stages in this species. Therefore, this study evaluated six candidate reference genes (*RPS2*, *ACTB*, *SDHA*, *B2M*, *UBE2G2*, and *RPL4*) based on prior transcriptomic sequencing of *P. roseus* blood. Using statistical algorithms (GeNorm, NormFinder, and BestKeeper) and the online tool RefFinder, we evaluated the expression stability of these genes in blood samples from females, males, and nestlings. During feather morphogenesis, *Ephrin-B1* (*EFNB1*) induces the formation of a ring-shaped expression domain at the base of feather bud, coordinating with the spatially specific expression of its receptor *EphB3* to jointly mediate cellular rejection signals [20]. This interaction is crucial for stabilizing the feather bud–interbud boundary. Functional inhibition of *Ephrin-B1* results in blurred bud boundaries, loose dermal condensation, and aberrant barb ridge patterns [21]. Distinct developmental boundaries are the foundation for the periodic arrangement of feathers. Their spatial precision ensures a uniform distribution of feathers across the body surface, preventing aerodynamic turbulence caused by uneven coverage. Closely arranged feathers form a continuous boundary layer that reduces airflow separation and vortex formation while significantly minimizing flight resistance [20]. For migratory birds, this energy-saving effect is particularly prominent, as it reduces flight energy consumption, extends continuous flight time, and avoids unnecessary weight gain [22,23]. Therefore, *Ephrin-B1* was selected as the target gene and compared with the above candidate reference genes through relative quantitative comparison to determine the optimal reference gene for the species. The results of the study will help in further in-depth analyses of the gene function of the *P. roseus*, aiming to provide a theoretical basis for the conservation and utilization of the species.

## 2. Materials and Methods

### 2.1. P. roseus Blood Collection and Preservation

In Yining County (44°04′ N, 81°60′ E, and altitude 978 m), Ili Kazakh Autonomous Prefecture, Xinjiang Uygur Autonomous Region, *P. roseus*, including females, males, and nestlings, were captured using mist nets. Blood samples were collected from the wing vein and transferred to cryotubes pre-treated with EDTA. The blood was gently pipetted 3–5 times to ensure thorough mixing with the EDTA solution. Subsequently, TRIzol reagent (Tiangen Biochemical Technology Co., Ltd., Beijing, China) was added at a blood-to-TRIzol ratio of 1:3. After vigorous vortexing for 30 s, samples were immediately flash-frozen in liquid nitrogen for preservation.

### 2.2. Total RNA Extraction and cDNA Synthesis

Total RNA was extracted from the blood of females, males, and nestlings (5 individuals per group) using the TRIzol method (Invitrogen, Waltham, MA, USA). The purity and concentration of total RNA were measured using the NanoDrop 2000 (Thermo Fisher, Waltham, MA, USA), and integrity was assessed via 1% agarose gel electrophoresis. Potential gDNA contamination was eliminated using the PrimeScript^TM^ RT Reagent Kit with gDNA Eraser (TaKaRa, Kusatsu, Japan), and first-strand cDNA was synthesized. cDNA is stored at −20 °C for subsequent use.

### 2.3. Primer Design and Standard Curve Establishment

Six candidate reference genes (*RPS2*, *ACTB*, *SDHA*, *B2M*, *UBE2G2*, and *RPL4*) were selected. Based on transcriptomic sequences, fluorescence quantitative primers were designed using Primer Premier 5.0 (Table 1) and synthesized by Sangon Biotech (Shanghai, China) Co., Ltd. Blood cDNA samples (extracted as described in Section 2.2) underwent serial dilution. RT-qPCR was performed to detect the *C*_t_ values of the six candidate reference genes. Standard curves were plotted to calculate the amplification efficiency (*E*) and correlation coefficients (*R*^2^) using the following equation: *E*(100%) = (10^(−1/slope)^ − 1) × 100. Each gradient was set up with three replications.

### 2.4. Quantitative Real-Time PCR

Using cDNA templates extracted from blood samples for female, male, and nestling *P. roseus*, relative expression levels of candidate reference genes were detected. Real-time fluorescence quantitative PCR was performed using the FQD-96C analyzer (Hangzhou, China) with the TB Green™ Premix Ex Taq™ II Kit (TaKaRa). Five biological samples were analyzed for each group (females, males, and nestlings), with each sample tested in triplicate as technical replicates. DEPC-treated water was used as the negative control. The 10 μL reaction mixture contained the following: 5 μL of 2× SYBR Green Master Mix, 0.4 μL each of forward and reverse primers, 1 μL of the cDNA template, and 3.2 μL of ddH_2_O. The reaction procedure was as follows: 95 °C for 30 s, 95 °C for 5 s, and 60 °C for 30 s (40 cycles). Fluorescence signals were collected from 62 °C to 95 °C every 6 s to plot the dissolution curve and evaluate primer specificity. The cDNA template was subjected to a 10-fold gradient dilution with six gradients, and the standard curves for each primer pair were plotted according to the fluorescence quantification results to calculate the amplification efficiency.

### 2.5. Stability Analysis of Reference Genes

Following RT-qPCR, cycle threshold (*C*_t_) values were collected and analyzed using three computational algorithms to evaluate reference gene stability: GeNorm, NormFinder (https://seqyuan.shinyapps.io/seqyuan_prosper/ accessed on 10 May 2025), and BestKeeper (https://www.gene-quantification.de/bestkeeper.html accessed on 10 May 2025) [24]. Finally, the online tool RefFinder (http://www.ciidirsinaloa.com.mx/RefFinder-master/ accessed on 20 May 2025) was used to integrate the results to generate a comprehensive stability ranking. Analyses using GeNorm and NormFinder software required the conversion of *C*_t_ values for each gene to 2^−Δ*C*t^, where —Δ*C*_t_ = *C*_t_ (each sample)—*C*_t_ (lowest sample). BestKeeper analyses use the average *C*_t_ value for each sample. BestKeeper software only requires the average *C*_t_ value of each sample. Lower M values calculated using GeNorm software, *SV*s (stability values) calculated using NormFinder software, and *CV* (coefficient of variation) and *SD* (standard deviation) values indicate more stable expression of the candidate reference gene.

### 2.6. Relative Expression Analysis of Ephrin-B1

Using the primers listed in Table 1, relative expression levels of *Ephrin-B1* were estimated. Using each of the six candidate reference genes as a standard, the 2^−ΔΔCT^ (Livak) method [25] was applied to analyze the mRNA expression levels of *Ephrin-B1*.

## 3. Results and Analysis

### 3.1. Total RNA Quality Control

All RNA samples were of good quality, as determined using the NanoDrop 2000, with A260/A280 ratios of 1.9–2.0 and A260/A230 ratios of approximately 2.0. The agarose gel electrophoresis results showed clear band patterns, indicating that the RNA samples maintained sufficient purity and integrity for subsequent experiments.

### 3.2. Validation of Primer Specificity and Amplification Efficiency for Candidate Reference Genes

**Figure 1:**
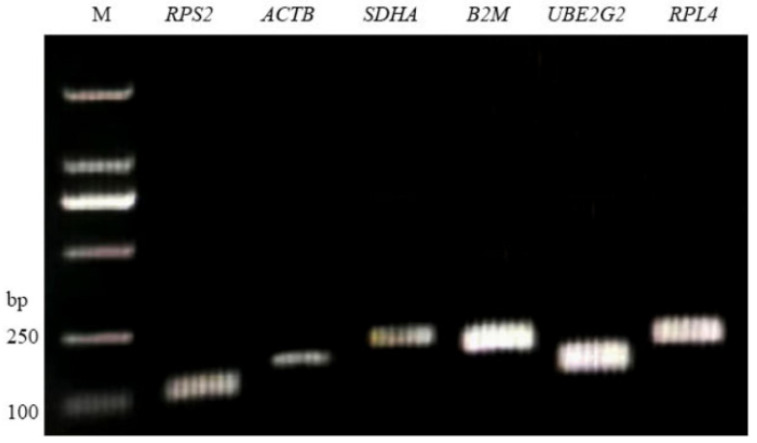
Real-time PCR results for six reference genes. Note: M: DNA marker DL2000.

**Figure 2:**
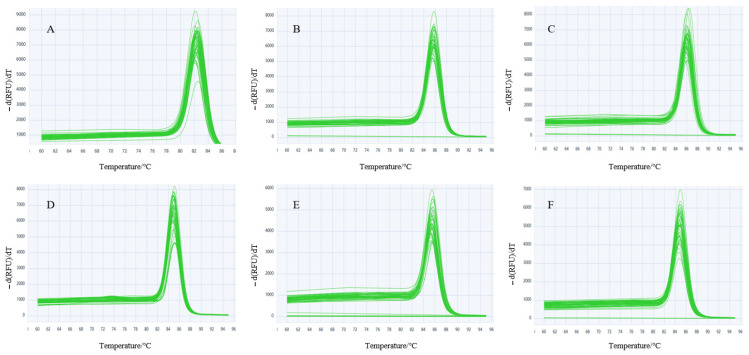
Melting curves of 6 reference genes. Note: (**A**–**F**) represent the melting curves for *RPS2*, *ACTB*, *SDHA*, *B2M*, *UBE2G2,* and *RPL4*, respectively.

Following the agarose gel electrophoresis of qPCR products, the amplified fragment lengths matched the expected sizes for all target genes. Single discrete bands were observed with no non-specific amplification or primer dimers (Figure 1), confirming primer specificity for the candidate reference genes. Amplification efficiencies were 94–113%, and correlation coefficients (*R*^2^) ranged from 0.9942 to 0.9982 for all six reference genes (Table 1). Each gene exhibited a single peak in the melt curve analysis (Figure 2), with no primer-dimer formation, indicating high reaction specificity and validating the effectiveness of these candidate reference genes for subsequent experiments.

### 3.3. Expression Stability of Candidate Reference Genes

**Figure 3:**
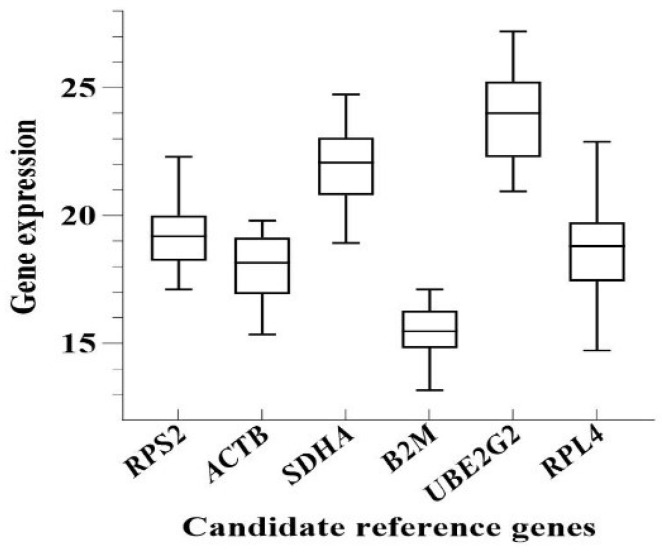
Expression levels of reference genes in females, males, and nestlings. Note: The expression levels of candidate reference genes were shown as *C*_t_ values. The upper and lower edges of the boxes represent the 75th and 25th percentiles, respectively. Whiskers represent the minimum and maximum *C*_t_ values. The line in the box represents the median.

As shown in box plots in Figure 3, expression levels of candidate reference genes varied across female, male, and nestling *P. roseus*, with *C*_t_ values of 13.17–27.21. Standard deviations, in decreasing order, were as follows: *RPL4* > *UBE2G2* > *SDHA* > *ACTB* > *RPS2* > *B2M*. Among these, *B2M* exhibited the highest expression stability, followed by *RPS2* and *ACTB*. The mean *C*_t_ values, *SD*, and *CV* were calculated using GraphPad Prism 8.0.1 (Table 2). As shown in Table 2, the expression abundance was highest for *B2M* (mean *C*_t_ = 15.42) and lowest for *UBE2G2* (mean *C*_t_ = 23.75). Based on *CV* values, *RPL4* was the least stable (*CV* = 10.09%), whereas *RPS2* (*CV* = 6.64%) and *B2M* (*CV* = 6.75%) demonstrated superior stability.

### 3.4. Comprehensive Stability Evaluation of Candidate Reference Genes

#### 3.4.1. GeNorm Analysis

**Figure 4:**
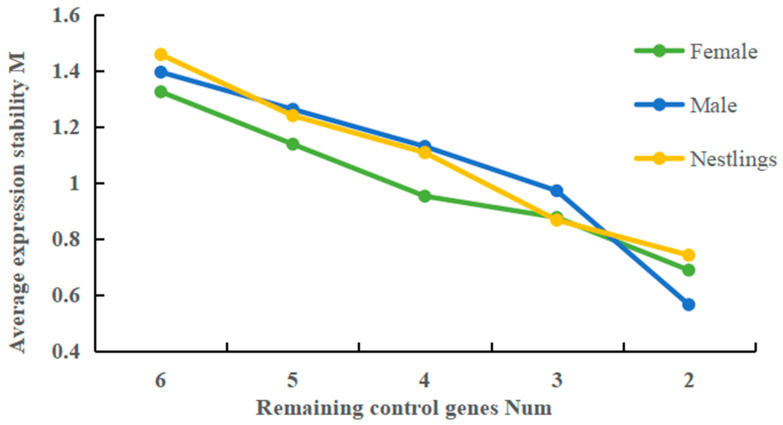
Impact of the number of control genes on the stability of average expression levels.

The average expression stability value (M) of each candidate reference gene was calculated using GeNorm software. The M value was evaluated through the stepwise exclusion of the reference genes with the lowest stability. Lower M values indicate higher stability. The six candidate genes in all groups (females, males, and nestlings) (Figure 4) exhibited progressively decreasing average M values, indicating enhanced gene stability. As shown in Table 3, in females, *ACTB* was the most stable and *B2M* was the least stable. In males, *RPS2* exhibited the highest stability, whereas *UBE2G2* ranked the lowest. In nestlings, *SDHA* was the most stable gene and *RPS2* was the least stable.

#### 3.4.2. NormFinder Analysis

**Figure 5:**
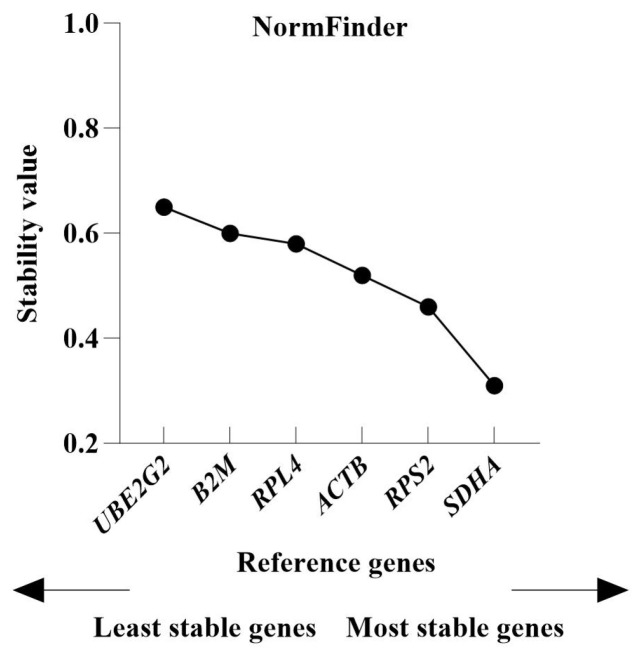
Stability of candidate reference gene expression determined using NormFinder software.

Unlike the GeNorm algorithm, the NormFinder software evaluates the stability of gene expression based on the stability value (*SV*), where a lower SV indicates greater gene stability. As shown in Figure 5, *SDHA* exhibited the lowest *SV* (0.31) and was identified as the most stable gene. *UBE2G2* had the highest *SV* (0.65) and was the least stably expressed gene. The six candidate reference genes were ranked in descending order of stability as follows: *SDHA* > *RPS2* > *ACTB* > *RPL4* > *B2M* > *UBE2G2*.

#### 3.4.3. BestKeeper Analysis

The *SD*, *CV*, and correlation coefficient (r) of each candidate reference gene were calculated using BestKeeper. Genes with *SD* < 1 were considered to have good expression stability. Lower *SD* and *CV* values, along with higher *r* values, indicate a greater stability (Table 4). Among the female, male, and nestling *P. roseus*, only *B2M* exhibited *SD* < 1, confirming its high stability. The ranking of SD values from high to low among the six reference genes was as follows: *B2M* > *RPS2* > *ACTB* > *SDHA* > *RPL4* > *UBE2G2*. The ranking of CV values from high to low was as follows: *B2M* > *RPS2* > *SDHA* > *ACTB* > *UBE2G2* > *RPL4*, and the ranking of r values from high to low was as follows: *SDHA* > *RPL4* > *ACTB* > *UBE2G2* > *RPS2* > *B2M*. All six genes showed statistical significance (*p* < 0.05), supporting that all six genes could be used in combination with other genes as co-reference genes.

#### 3.4.4. RefFinder Analysis

Results obtained using GeNorm, NormFinder, and BestKeeper algorithms exhibited a broad consensus, with *SDHA*, *ACTB*, and *B2M* demonstrating high stability across all analyses. However, there were minor differences in exact rankings. To resolve these discrepancies, RefFinder was used to integrate outputs from all three algorithms, generating the following consolidated stability ranking: *SDHA* > *ACTB* > *RPS2* > *B2M* > *RPL4* > *UBE2G2* (Table 5).

**Figure 6:**
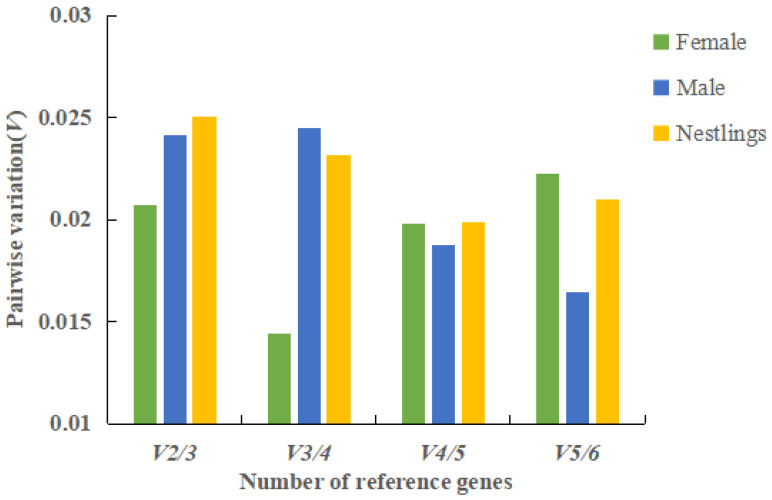
Pairwise variation (V) analysis of the six candidate reference genes in all tested samples.

In addition, the minimum number of reference genes required for reliable normalization was determined by calculating pairwise variation (V*_n_*_/*n*+1_) using GeNorm software. When V*_n_*_/*n*+1_ < 0.15, *n* reference genes are sufficient to correct the data; otherwise, *n* + 1 reference genes are required. In this study, all groups (females, males, and nestlings) exhibited V_2/3_ < 0.025 (Figure 6), indicating that two reference genes were sufficient for RT-qPCR data normalization.

#### 3.4.5. Validation of Reference Gene Expression Stability

**Figure 7:**
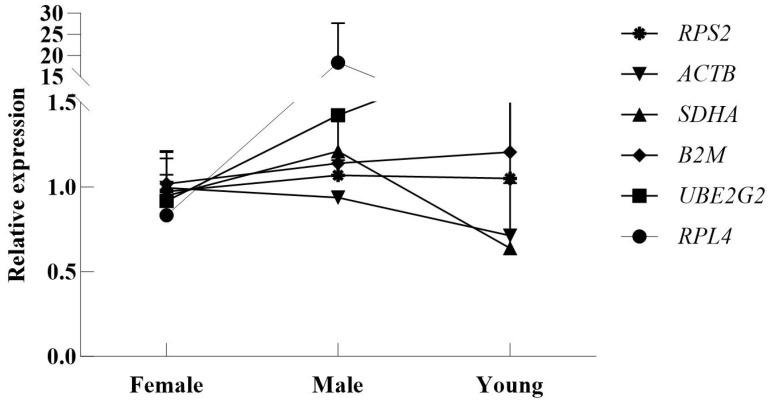
Relative quantification of *Ephrin-B1* gene expression using various reference genes for normalization.

In this study, the relative expression levels of the target gene *Ephrin-B1* were quantified using six distinct candidate reference genes for normalization. The results revealed significant variation in *Ephrin-B1* expression across experimental conditions (Figure 7).

## 4. Discussion

Xinjiang hosts a diverse array of locust species with extensive distributions and staggering population densities. Annually, locust infestations affect over 2 × 10^6^ hm^2^ of grasslands, inflicting severe economic losses on agriculture and animal husbandry in the region [26]. *P. roseus* serves as an effective biological control agent against locust infestations in Xinjiang’s grasslands, suppressing locust outbreaks significantly through its exceptional predation capacity [11]. Given the scarcity of molecular biology research on *P. roseus*, systematically characterizing the expression profiles of migration- and metabolism-related genes and their regulatory pathways holds significant scientific value for deciphering molecular adaptations underlying long-distance migration and dynamic energy regulation. The quantification of gene expression levels is a prerequisite for analyzing biological functions. As a robust technique for gene expression analyses, quantitative real-time PCR is used extensively in functional genomics research [27] and has emerged as a pivotal tool for profiling candidate gene expression signatures [28]. In this process, the selection of reference genes is critical. Based on specific experimental conditions (e.g., tissue type, treatment factors, and developmental stage), stable reference genes must be rigorously screened for normalization. This enables the precise quantification of target gene expression dynamics [29] and the generation of reliable data for in-depth functional interpretation [30].

Succinate dehydrogenase flavoprotein subunit A (*SDHA*) has a fundamental role in energy metabolism and has been validated as a reliable reference under specific conditions [31]. A screening study of reference genes in nine bird species, including *Anas platyrhynchos*, *Gallus gallus domesticus*, and *Struthio camelus*, revealed that *SDHA* exhibited high conservation across avian species and was suitable for cross-species qPCR studies [32]. The stability of *SDHA* exhibits significant differences in tissue-specific expression. For example, in the liver, thymus, and intestine of ducks, the stability of *SDHA* is moderate, requiring combination with other reference genes (e.g., 18S rRNA) to enhance standardization effects. In contrast, in the reproductive axis tissues of pigeons, the stability of *SDHA* is significantly higher than that of *ACTB*, and the *B2M* gene demonstrates the most stable expression [16]. Additionally, in the small intestine of juvenile mice [33] and in adult male *Lygus pratensis* [34], *SDHA* exhibits the highest expression stability, making it suitable as a reference gene. This indicates that the selection of appropriate reference genes must be combined with species, tissue, and physiological state differences for comprehensive analysis. In the study of *P. roseus*, analysis using the GeNorm software revealed that the stability of *SDHA* varies across different genders and developmental stages, potentially related to hormonal levels and physiological states. Although *SDHA* exhibits poor stability in *Oxygymnocypris stewartii* and may not be suitable for fish studies [35], it remains reliable in energy metabolism-related research. As a migratory bird, the high energy demands of *P. roseus* further support the suitability of *SDHA as* an internal reference gene.

*ACTB* (also known as the β-actin gene) is a common housekeeping gene that encodes β-actin, a core component of the cytoskeleton. It participates in the maintenance of cellular morphology, signal transduction, and motility. Due to its significant role in cell structure and function, *ACTB* is widely used as a reference gene in real-time quantitative PCR [36]. It was identified as the most stably expressed internal reference gene in the small intestinal mucosa of Hu sheep [37], the skeletal muscle of porcine [38], and *Larimichthys crocea* [39]. In experiments on fructose-fed rats, the expression of *ACTB* in the liver was significantly elevated in specific groups (fasted fructose-fed), which might affect standardization results [40]. Additionally, during the screening of reference genes in the brain tissues of songbirds and other birds, it was clearly indicated that *ACTB*, commonly used in mammals, is not suitable for use in the gonads and brain tissues of birds [41,42].

Ribosomal proteins (RPs) refer to the proteins that constitute ribosomes. Based on subunit size, RPs can be categorized into ribosomal large subunit proteins (RPL) and ribosomal small subunit proteins (RPS) [43]. *RPS2*, a ribosomal protein-coding gene, was identified alongside *GluR2* and *18S rRNA* as the most stable reference gene combination in domestic pigeons. Its expression stability (M < 0.5) was significantly greater than those of *ACTB* and *GAPDH*, demonstrating suitability for crossbreed comparative expression analyses (e.g., analyses of the magnetoreception gene *Cry4*) [15]. Furthermore, the combination of *RPS2* + *18S rRNA* has been validated as the optimal reference gene pair for gene expression studies across diverse tissues in *C. livia* [12]. The screening of reference genes in *Gallus gallus domesticus* under different concentrations revealed that *RPS2* and *TBP* exhibited a higher gene expression stability [44]. In *Taeniopygia guttata*, *B2M* showed stable primer amplification efficiency (95–105%), making it suitable for multi-species comparisons [32]. In this study, the BestKeeper algorithm identified *B2M* as the optimal reference gene for *P. roseus* under various conditions. However, in other algorithms, the ranking of *B2M* was consistently lower, indicating that the variability in its stability may reflect a heightened sensitivity to different algorithmic approaches in evaluating reference genes. This variability may arise from differences in algorithm design, data processing methods, and experimental conditions. Therefore, to enhance the reliability of selecting the optimal reference gene for *P. roseus*, it is advisable to cross-validate results across multiple algorithms.

In experiments on the infectious laryngotracheitis virus (ILTV) in chickens, *RPL4* was selected as a reference gene [45]. *RPL4* has also demonstrated high stability in studies on insects (e.g., aphids and beetles) and fungi (e.g., *Ganoderma lucidum*) [46]. However, in this study, when selecting the optimal reference genes for *P. roseus* across different genders and developmental stages using various algorithms, *RPL4* consistently ranked relatively low. Current studies have not fully compared the stability of *RPL4* in birds across different species or conditions. Therefore, whether *RPL4* can serve as an optimal reference gene for *P. roseus* requires further investigation. Currently, there is no report in avian studies on the use of *UBE2G2* as a reference gene.

Based on the transcriptome data of *P. roseus* blood obtained in a previous study, reference genes were selected as candidate internal reference genes in this study, and their expression stability was evaluated in female, male, and nestling *P. roseus*. The three analysis software, GeNorm, NormFinder, and BestKeeper, all indicated that the expression stability of *SDHA*, *ACTB*, and *RPS2* in *P. roseus* was relatively high. However, due to the different algorithms of the software, the stability ranking of the internal reference genes varied slightly. GeNorm identified *SDHA/ACTB* as the most stable pair, NormFinder ranked *SDHA as* the top gene, and BestKeeper identified *B2M* as the most stable gene. GeNorm analysis of pairwise variation values (V*_n_*_/*n*+1_) revealed that the optimal number of reference genes for females, males, and nestlings was two. RefFinder, the only web-based tool for comparing and evaluating reference genes, integrates four calculation programs (GeNorm, NormFinder, BestKeeper, and the comparative Δ*C_t_* method) into a web-based tool to evaluate the stability and reliability of reference genes. Based on the stability rankings generated by these four programs, appropriate weights are assigned to each gene, and the geometric mean of the weighted ranking is calculated to determine the final overall ranking. In addition to the overall ranking, individual programs or combinations of the four programs can also be selected to evaluate the ranking of candidate reference genes [47]. Given the discrepancies among the results generated by GeNorm, NormFinder, and BestKeeper, RefFinder was finally used in this study to establish the comprehensive stability of the candidate internal reference genes. Results from RefFinder indicate that the optimal combination of reference genes for *P. roseus* is *SDHA*/*ACTB*, as calculated by RefFinder, NormFinder, and RefFinder. The GeNorm software identified the optimal combination as *SDHA/RPL4*, while BestKeeper software determined the optimal combination as *B2M/RPS2*. These results demonstrate that RefFinder is capable of evaluating and selecting optimal reference genes, as its findings align with the results from different algorithms [48].

Accordingly, in this study, *Ephrin-B1* was selected as the target gene for quantitative expression analyses to validate reference gene stability. Relative quantification of *Ephrin-B1* using six candidate reference genes revealed significant variation in measured expression levels, confirming that the stability of reference genes indeed affects the accuracy of RT-qPCR results and leads to incorrect analytical conclusions. A comprehensive analysis revealed that the best reference gene combination for *P. roseus* was the *SDHA/ACTB* gene pair. The research results provide a basis for a wide range of studies, e.g., elucidating gene regulatory networks underlying seasonal reproduction in *P. roseus*, reliably detecting interpopulation expression differences, deciphering genetic mechanisms underlying migratory behavior regulation, and identifying key genes and molecular pathways in energy metabolism.

## 5. Conclusions

In this study, based on different algorithmic approaches and literature reports, we recommend using two reference genes for the normalization of RT-qPCR data in *P. roseus*. Specifically, the combination of *SDHA*/*ACTB* is suggested as the optimal reference gene pair for blood-related studies in *P. roseus*. These findings provide a foundation for future research on the regulatory mechanisms of gene expression in *P. roseus*. Future studies could further explore the transcriptomic and epigenetic regulatory mechanisms, as well as the expression characteristics of migration and energy metabolism-related genes in *P. roseus*. This research could contribute to the effective control of grasshopper populations, ecological balance, and biodiversity conservation.

## Figures and Tables

**Table 1:** The primers used for RT-qPCR.

Gene Symbol	Gene Name	Primer Sequences (5′-3′)	Product Size (bp)	GenBank ID	Standard Curve	*E* (%)	*R* ^2^
*RPS2*	Ribosomal Protein S2	F:GGAGTGGATTCCTGTCACC	130	PV870197	y = −3.2377x + 19.031	104	0.9979
		R:CGAGGAGCCCAGGAAGAAATC					
*ACTB*	β-Actin	F:CATCTACGAAGGCTATGCCC	142	PV870193	y = −3.0362x + 16.948	113	0.9982
		R:GTCACGCACGATTTCTCTCTC					
*SDHA*	Succinate Dehydrogenase Complex Subunit A	F:CGCAATACCCAGTAGTGGACC	153	PV870198	y = −3.1911x + 21.564	106	0.9969
		R:GTGCAGCAACAGTATGAGAGCG					
*B2M*	β-2-microglobulin	F:CGAGGAGGGAAAGGAGAAC	170	PV870194	y = −3.0703x + 14.255	112	0.9942
		R:GGATGAAGGGCACATAGACC					
*UBE2G2*	Ubiquitin Conjugating Enzyme E2G 2	F:TCCATCCTTCACGCTCCT	133	PV870199	y = −3.4677x + 20.254	94	0.9963
		R:CACTTTCATCATTTGGCTCT					
*RPL4*	Ribosomal Protein L4	F:CCAACCTGCGCAAGAACAAC	173	PV870196	y = −3.1957x + 19.031	106	0.9948
		R:GCCGCCACGACACATATTG					
*EFNB1*	Ephrin-B1	F:TCAAGTTCCAGGAGTTCAGCC	98	PV870195			
		R:GCCATCCAGCGTGCCATT					

**Table 2:** Analysis of values and expression of candidate reference genes.

Gene	Mean Quantification Cycle (*C*_t_)	*SD*	Coefficient of Variation (*CV* %)
*RPS2*	19.25	1.28	6.64%
*UBE2G2*	23.75	1.77	7.45%
*SDHA*	21.88	1.49	6.80%
*RPL4*	18.73	1.89	10.09%
*ACTB*	17.98	1.30	7.24%
*B2M*	15.42	1.04	6.75%

**Table 3:** Ranking of internal reference genes in different groups.

Rank	Female	Male	Nestlings
1	*ACTB*	*RPS2*	*SDHA*
2	*SDHA*	*ACTB*	*RPL4*
3	*RPS2*	*SDHA*	*ACTB*
4	*UBE2G2*	*B2M*	*B2M*
5	*RPL4*	*RPL4*	*UBE2G2*
6	*B2M*	*UBE2G2*	*RPS2*

**Table 4:** Stability analysis of the candidate reference genes by BestKeeper.

Genes	*N*	Geometric Mean [*C*_t_]	Arithmetic Mean [*C*_t_]	Min [*C*_t_]	Max [*C*_t_]	*SD* [ ± *C*_t_]	*CV* [%*C*_t_]	*r*	*p*
*RPS2*	15	19.21	19.25	17.28	22.21	1.01	5.26	0.668	0.006
*ACTB*	15	17.93	17.98	15.53	19.66	1.10	6.09	0.813	0.001
*SDHA*	15	21.83	21.88	19.08	24.72	1.22	5.55	0.938	0.001
*B2M*	15	15.39	15.42	13.50	16.77	0.8	5.17	0.602	0.001
*UBE2G2*	15	23.69	23.75	21.24	26.51	1.49	6.29	0.749	0.001
*RPL4*	15	18.64	18.73	14.98	22.69	1.35	7.21	0.865	0.001

**Table 5:** Expression stability ranks of the candidate reference genes evaluated by RefFinder.

Method	Expression Stability Ranks					
	1	2	3	4	5	6
Delta CT	*SDHA*	*ACTB*	*RPS2*	*B2M*	*RPL4*	*UBE2G2*
Genorm	*SDHA*/*RPL4*		*ACTB*	*RPS2*	*B2M*	*UBE2G2*
Normfinder	*SDHA*	*ACTB*	*RPS2*	*B2M*	*RPL4*	*UBE2G2*
BestKeeper	*B2M*	*RPS2*	*ACTB*	*SDHA*	*RPL4*	*UBE2G2*
RefFinder	*SDHA*	*ACTB*	*RPS2*	*B2M*	*RPL4*	*UBE2G2*

## Data Availability

The reference genes used in this study were derived from our team’s original transcriptome data, which have been deposited in the NCBI Sequence Read Archive (SRA) and are accessible via the accession number: PRJNA1281702. These data will be made publicly at an appropriate time.
